# NLRP3 Inflammasome Activation by *Paracoccidioides brasiliensis*

**DOI:** 10.1371/journal.pntd.0002595

**Published:** 2013-12-05

**Authors:** Aldo Henrique Tavares, Kelly Grace Magalhães, Raquel Das Neves Almeida, Rafael Correa, Pedro Henrique Burgel, Anamélia Lorenzetti Bocca

**Affiliations:** 1 Faculdade de Ceilândia, Universidade de Brasília, Brasília, Distrito Federal, Brasil; 2 Laboratorio de Imunologia Aplicada, Departamento de Biologia Celular, Instituto de Biologia, Universidade de Brasília, Brasília, Distrito Federal, Brasil; 3 Laboratorio de Imunologia e Inflamação, Departamento de Biologia Celular, Instituto de Biologia, Universidade de Brasília, Brasília, Distrito Federal, Brasil; University of California San Diego School of Medicine, United States of America

## Abstract

*Paracoccidioides brasiliensis* is the etiologic agent of paracoccidioidomycosis (PCM), the most prevalent systemic mycosis that is geographically confined to Latin America. The pro-inflammatory cytokine IL-1β that is mainly derived from the activation of the cytoplasmic multiprotein complex inflammasome is an essential host factor against opportunistic fungal infections; however, its role in infection with a primary fungal pathogen, such as *P. brasiliensis*, is not well understood. In this study, we found that murine bone marrow-derived dendritic cells responded to *P. brasiliensis* yeast cells infection by releasing IL-1β in a spleen tyrosine kinase (Syk), caspase-1 and NOD-like receptor (NLR) family member NLRP3 dependent manner. In addition, *P. brasiliensis*-induced NLRP3 inflammasome activation was dependent on potassium (K+) efflux, reactive oxygen species production, phagolysosomal acidification and cathepsin B release. Finally, using mice lacking the IL-1 receptor, we demonstrated that IL-1β signaling has an important role in killing *P. brasiliensis* by murine macrophages. Altogether, our results demonstrate that the NLRP3 inflammasome senses and responds to *P. brasiliensis* yeast cells infection and plays an important role in host defense against this fungus.

## Author Summary

Paracoccidioidomycosis is a systemic disease that has an important mortality and morbidity impact in Latin America. It mainly affects rural workers of Argentina, Colombia, Venezuela and Brazil. Upon host infection, one of the most important aspects that contribute to the disease outcome is the initial interaction of the *Paracoccidioides brasiliensis* fungus with the phagocytic cells and the induction of the inflammatory process. Among several inflammatory mediators, the cytokine interleukin-1β is of pivotal importance in this complex process. Here, we demonstrate that *P. brasiliensis* is sensed by the NLRP3 inflammasome, a cytoplasmatic multiprotein complex that lead to the processing and secretion of IL-1β. In addition, we described the intracellular perturbations that may be associated with NLRP3 activation such as potassium efflux, production of reactive oxygen species, and lysosomal damage. Finally, our work provides evidence for the protective role of IL-1β during fungal infection of murine macrophages.

## Introduction

*Paracoccidioides brasiliensis* is a thermally dimorphic fungus that causes paracoccidioidomycosis (PCM), a systemic granulomatous mycosis that is endemic to South America, especially Brazil, Argentina, Venezuela and Colombia [1], [2]. In Brazil, PCM is the tenth most common fatal chronic infectious disease and the deadliest deep mycosis that is not associated with AIDS [3]. *P. brasiliensis* grows as saprophytic mycelia that produce infective conidia propagules, which are inhaled into the lungs where the fungus transitions to the pathogenic yeast form. This step is essential for the successful establishment of infection [4]–[6].

Once in the lungs, innate immune cells, such as resident macrophages and dendritic cells, are the first line of defense that interact with *P. brasiliensis* cells [7]. Such activity is based on the recognition of conserved microbial structures, known as pathogen-associated molecular patterns (PAMPs), by germline-encoded pattern recognition receptors (PRRs) [8], [9]. In particular, the Toll-like receptors (TLRs) TLR-2, TLR-4 and TLR-9 as well as the C-type lectin receptor (CLR) dectin-1 play a role in the recognition of *P. brasiliensis* and the modulation of the immune response [10]–[15]. The recognition of PAMPs by these PRRs leads to the secretion of pro-inflammatory cytokines, including TNF-α, IL-6 and IL-1β, which is mainly dependent on NFκB-promoted transcription [7], [16]. Remarkably, IL-1β is essential to the inflammatory response to infections and is not released via the classical endoplasmic reticulum-Golgi secretory pathway. IL-1β is retained in the cytoplasm as an inactive form, known as pro-IL-1β, which is proteolytically processed into the 17-kDa biological form by the cysteine protease caspase-1 and then secreted by a poorly characterized, unconventional protein secretion pathway [17]. Like other caspases, caspase-1 itself is produced as a pro-enzyme, and its activation has been associated with nucleotide binding and oligomerization domain (NOD)-like receptors (NLRs), an extensive family of cytosolic PRR. Pro-caspase-1-processing NLRs form a cytoplasmic multiprotein complex called the inflammasome, which includes the adaptor protein ASC (apoptosis-associated speck-like protein containing a C-terminal caspase recruitment domain, CARD) [18], [19]. Among the caspase-1-activating inflammasomes described [18], the most comprehensively studied is the NOD-like receptor family, pyrin domain containing 3 (NLRP3) inflammasome, which is activated by diverse stimuli, including microbial products (e.g., muramyl dipeptides) and endogenous danger signals [18], [19]. Upon activation, NLRP3 recruits ASC, which in turn recruits pro-caspase-1 to assemble the NLRP3 inflammasome, which leads to caspase-1 activation [18], [19], [20]. In this manner, two separate signals control IL-1β release. The first priming signal relies on PAMP recognition by PRRs, which directs pro-IL-1β transcription and translation via NFκB. In contrast, the second activation signal, which is usually derived from danger signals, involves pro-IL-1β cleavage by inflammasome-dependent caspase-1 into a mature cytokine. Endogenous danger signals include extracellular ATP, phagolysosomal damage, reactive oxygen species (ROS) production and the induction of transmembrane ion fluxes [19], [20].

The NLRP3 inflammasome senses several pathogens, including bacteria, viruses and parasites [18]. Regarding fungi, the opportunistic pathogens *Candida albicans*, *Aspergillus fumigatus*, *Cryptococcus neoformans* and *Trichophyton schoenleinii* induce IL-1β release via the NLRP3 inflammasome [21]–[26]. Notably, IL-1β signaling via its receptor IL-1R1 (interleukin 1 receptor, type I) and NLRP3 inflammasome components are essential for host defense against *C. albicans* and *C. neoformans* [21], [23], [25]. Considering the role of the NLRP3 inflammasome in fungal infections and that IL-1β release has been detected in phagocytes and mice infected with *P. brasiliensis* [27]–[30], we aimed to evaluate whether this primary fungal pathogen induces IL-1β production via inflammasome-dependent caspase-1 activation in murine macrophages and dendritic cells. In this study, we demonstrated that IL-1β production induced by the yeast cells of *P. brasiliensis* is dependent on NLRP3 and caspase-1 activity in dendritic cells. In addition, potassium (K^+^) efflux, ROS production, lysosomal acidification and cathepsin B release are required for the activation step of the inflammasome. Finally, we demonstrated the importance of IL-1β signaling in controlling *P. brasiliensis* intracellular growth in macrophage cells.

## Methods

### Ethics statement

All work was conducted with the approval of the Committee on the Ethics of Animal Experiments of the University of Brasilia (CEUA/UnB permit number: 54412/2011) according to the National Council on Animal Experiments and Control (CONCEA-MCT-Brazil) guidelines.

### Mice

C57BL/6 isogenic mice (8–10 weeks old) deficient in NLRP3 (NLRP3^−/−^) or IL-1R1 (IL-1R1^−/−^) and strain-matched wild type (WT) controls were used in this investigation. Gene-deleted mice were kindly provided by Prof. Dario S. Zamboni from University of São Paulo, Ribeirão Preto, São Paulo, Brazil. Mice were housed with food and water *ad libitum*.

### Fungus

The yeast form of the highly virulent *P. brasiliensis* isolate 18 was grown on Fava-Netto semisolid medium (0.3% protease peptone, 1% peptone, 0.5% beef extract, 0.5% yeast extract, 4% glucose, 0.5% NaCl and 1.6% agar, pH 7.2) for 7 days at 37°C before the *in vitro* infection experiments. Viability was determined using Janus Green B vital dye (Merck) and was always greater than 80%. To maintain its virulence, the isolate was used after serial animal passages.

### Generation of bone marrow-derived macrophages (BMDMs) and dendritic cells (BMDCs)

Bone marrow-derived cells were obtained as previously reported [31]. Briefly, femurs and tibias were flushed with RPMI-1640 to release the bone marrow cells. After erythrocyte lysis, the bone marrow cells (2×10^5^ cells/ml) were seeded and cultured for 8 days at 37°C in 9-cm non-tissue culture-treated Petri dishes in 10 ml/dish of RPMI-1640 medium that contained 50 µM 2-mercaptoethanol. The medium was supplemented with 20 ng/ml murine granulocyte-macrophage colony-stimulating factor (GM-CSF, Peprotech) or 30% conditioned medium from macrophage colony-stimulating factor-secreting L929 fibroblasts (M-CSF) to obtain BMDCs and BMDMs, respectively. On day 3, another 10 ml of fresh complete medium that contained differentiation-inducing cytokines was added to the culture. On day 6, only for the BMDC cultures, half of the medium was changed. On day 8, non- and loosely adherent BMDCs or firmly adherent BMDMs were harvested and plated in complete RPMI medium for experimental use. Flow cytometry evaluation indicated that those cells cultured in GM-CSF were 81% positive for CD11c and MHC class II, whereas the M-CSF cells were 91% positive for CD11b and 84% positive for F4/80 (data not shown).

### Infection of murine cell cultures with *P. brasiliensis* and treatments

BMDM and BMDC monolayers derived from WT or knockout mice were infected with 1×10^6^ *P. brasiliensis* yeast cells in 24-well culture plates, which represented a yeast-to-cell ratio of 1∶1 (multiplicity of infection, MOI: 1) as previously reported [14]. MOIs that are higher than 1 lead to murine cell death (data not shown). Incubation with the fungus was conducted at 37°C in a humidified 5% CO_2_ atmosphere for 24 h for both cytokine quantification and fungicidal assays. Additionally, we tested a 6 h incubation time, but IL-1β production peaked at the 24 h time point. Using an MOI of 1, an average of 60% of the BMDCs and 50% of the BMDMs were engaged in phagocytosis of at least one yeast cell by 6 h of incubation, which did not change significantly at 24 h (data not shown) [14]. In some experiments, the phagocytic cells were pre-treated (for 1 h) with either the NF-κB inhibitors Bay11-7082 (5 µM) and celastrol (5 µM) (both from InvivoGen), the cathepsin B inhibitor CA-074Me (50 µM) (Sigma-Aldrich), the endosomal acidification inhibitor bafilomycin (250 nM) (InvivoGen), the ATP-sensitive K^+^ channel inhibitor glibenclamide (150 µM) (InvivoGen), KCL (100 mM) (Sigma-Aldrich), ROS scavengers/inhibitors NAC (N-acetyl-L-cysteine) (20 mM) or APDC (2R,4R)-4 aminopyrrolidine-2,4-dicarboxylic acid) (100 µM) (both from Sigma-Aldrich), the caspase-1 inhibitor AC-Y-VAD-CHO (50 µM) (Santa Cruz Biotechnology), the caspase-8 inhibitor Z-IETD-FMK (50 µM) (R&D Systems), the Syk inhibitor piceatannol (3,4′,3′,5-trans-trihydroxystilbene) (25 µM) (InvivoGen) or the Myd88 inhibitory peptide Pepinh-MYD (50 µM) (InvivoGen). Inhibitors that required reconstitution were dissolved in distilled water, PBS or dimethyl sulfoxide (DMSO). Regarding DMSO, an equivalent quantity of this vehicle was added to the appropriate controls. In addition, the trypan blue exclusion test was used to evaluate cell toxicity in the inhibitor assays; however, no significant cell damage was observed (data not shown). In some experiments, the cells were treated with LPS (*Escherichia coli* serotype O111:B4) (100 ng/ml) (Sigma-Aldrich) and/or ATP (5 mM) (InvivoGen). The treatment with LPS and ATP was used as a positive control for NLRP3-mediated inflammasome activation. Following 1 h of ATP treatment, the medium supernatant was collected to measure the cytokine protein levels by ELISA.

### Cytokine measurements by enzyme-linked immunosorbent assay (ELISA)

The cell-free supernatants of the BMDM and BMDC cultures were harvested and stored at −20°C until the determination of the IL-1β, TNF-α (DuoSet kit, R&D Systems) and IL-6 (Ready-Set-Go! Kit, eBioscience) concentrations using ELISA. The determination of intracellular pro-IL-1β was performed after discarding the supernatant and lysing the cell monolayer by 2–3 freeze-thaw cycles. The cytokine quantification was performed according to the manufacturer's instructions. The data were expressed as pg/ml ± the standard deviation (SD) of two to three independent experiments, which were conducted in triplicate.

### Fungicidal assay

BMDMs from WT, NLRP3−/− and IL1R1−/− C57BL/6 mice were infected with *P. brasiliensis* and treated, or not, with ATP as described above. In addition, WT macrophages were also treated with IFN-γ (100 U/ml), as a control of the fungicidal activation. The number of viable fungi in the cell cultures was determined using colony-forming unit (CFU) evaluation. Briefly, after the culture supernatants were removed for nitric oxide determination, extracellular and weakly adherent fungi were removed by washing with pre-warmed RPMI. Macrophages were then lysed with distilled water. One hundred microliters of the cellular suspensions and serial dilutions were plated on brain-heart infusion agar (BHI, Difco), which was supplemented with 4% horse serum and 5% *P. brasiliensis* isolate 192 culture filtrate. The latter constitutes a source of growth-promoting factor. The plates were incubated at 37°C, and the colonies were counted after 5 days. The data were expressed as CFU/ml ± the SD of one experiment that was representative of three experiments, which were performed in triplicate.

### Nitric oxide production by BMDMs

Nitric oxide (NO) production was measured by the accumulation of nitrite in the supernatants of macrophages cultures using Griess assay. Briefly, 100 µl of the supernatants collected were mixed with an equal volume of Griess reagent (1% sulfanilamide, 0,1% naphthylene diamine dihydrochloride, 2,5% H_3_PO_4_) and 10 min later absorbance at 550 nm was determined. The nitrite concentration was determined in reference to a standard curve of NaNO_2_ diluted in RPMI medium. All determinations were performed in triplicates and expressed as micromolar NO_2_. As a control, macrophages infected with *P. brasiliensis* were treated or not with 100 U/ml of IFN-γ.

### Statistical analyses

GraphPad Prism 5.0 (GraphPad Software) was used for the statistical analyses. A paired two-tailed Student's *t*-test was used, and a *p* value ≤0.05 was considered statistically significant. In addition, multiple group comparisons were conducted using one-way ANOVA, followed by Bonferroni tests as appropriate.

## Results

### *P. brasiliensis* yeast cells induce inflammasome activation in BMDCs but not in BMDMs

**Figure 1:**
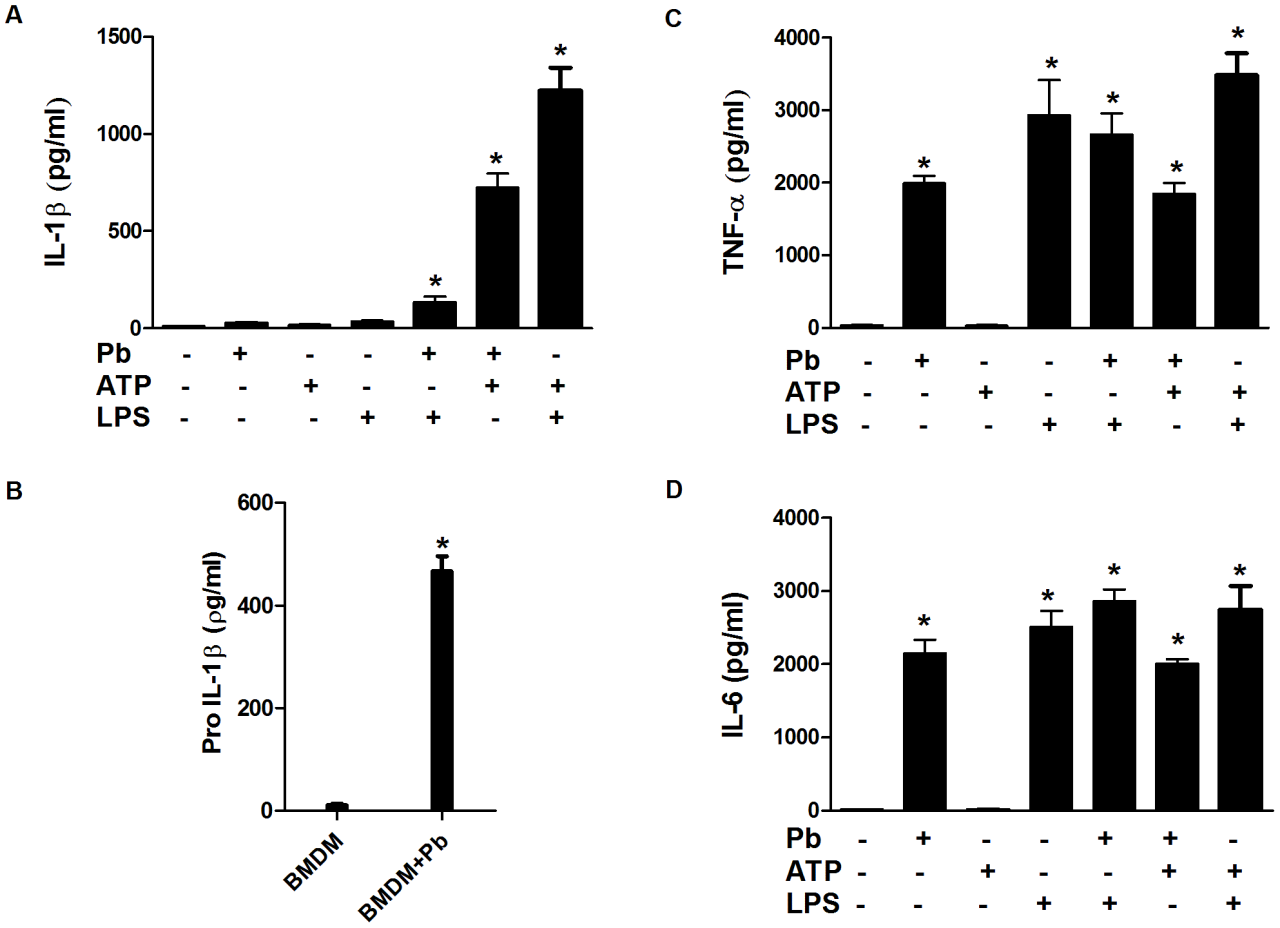
*P. brasiliensis* does not activate the inflammasome but induces the production of pro-IL-1β in BMDMs. BMDMs were stimulated for 24*P. brasiliensis* (Pb) (MOI 1), ATP, LPS, LPS and Pb (100 ng/ml for 2 h before fungal infection), Pb and ATP (the latter added 1 h before co-culture incubation time completion). As a control, BMDMs were primed with LPS, followed by ATP, as previously described. The supernatants from the cultures were harvested for (A) IL-1β, (C) TNF-α and (D) IL-6 assays using ELISA. (B) Pro-IL-1β was assayed in the cell lysate of BMDCs that were infected or not with *P. brasiliensis* for 24 h. The data are expressed as the mean ± the SD of two to three independent experiments conducted in triplicates. *denotes *p*≤0.05 compared with not infected cells.

**Figure 2:**
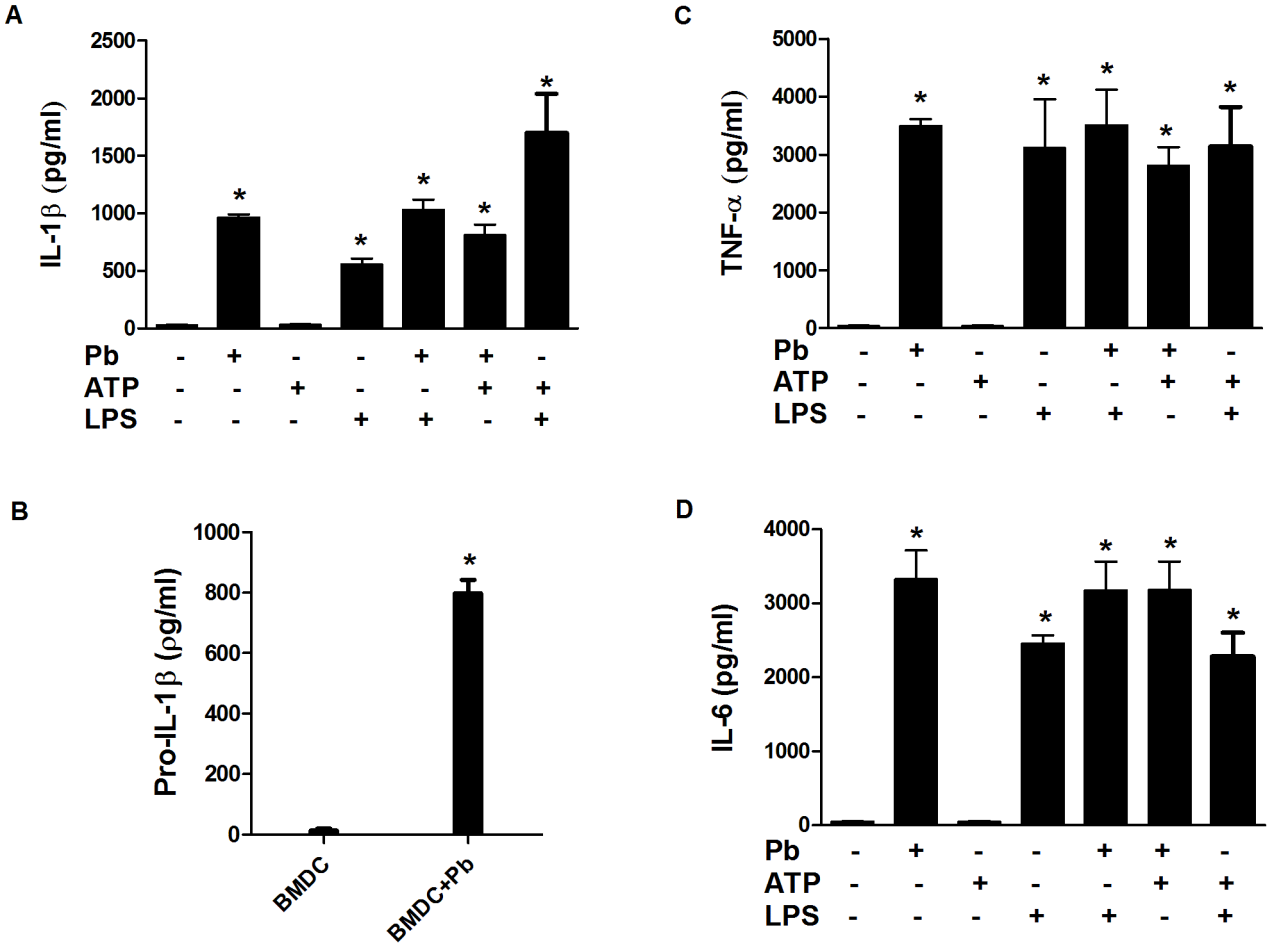
*P. brasiliensis* induces the production of pro-IL-1β and activates the inflammasome in BMDCs. BMDCs were stimulated for 24*P. brasiliensis* (Pb) (MOI 1), ATP, LPS, LPS and Pb (100 ng/ml for 2 h before fungal infection), Pb and ATP (added 1 h before co-culture incubation time completion). As a control, BMDMs were primed with LPS, followed by ATP, as previously described. The supernatants from the cultures were harvested for (A) IL-1β, (C) TNF-α and (D) IL-6 assays using ELISA. (B) Pro-IL-1β was assayed in the cell lysate of BMDCs that were infected or not with *P. brasiliensis* for 24 h. The data are expressed as the mean ± the SD of two to three independent experiments conducted in triplicates. *denotes *p*≤0.05 compared with not infected cells.

To investigate whether *P. brasiliensis* could induce IL-1β secretion, murine BMDMs and BMDCs were infected with *P. brasiliensis* yeast cells for 24 h, and mature IL-1β production was measured in the culture supernatants using ELISA. The fungus alone did not trigger IL-1β release in the BMDMs. In addition, priming with LPS (100 ng/ml for 2 h) before the fungal infection only poorly induced IL-1β production (Figure 1A). IL-1β release requires pro-IL-1β synthesis, followed by its processing; therefore, the lack of secretion of this cytokine may have resulted from the incapacity of *P. brasiliensis* to induce pro-IL-1β or cytokine maturation. To discriminate between these possibilities, we verified pro-IL-1β production. Pro-IL-1β was detected in the cell lysate using ELISA and was clearly produced in response to the fungal infection alone (Figure 1B). In addition, a robust production of mature IL-1β was observed in infected BMDMs only after treatment with ATP, which is a specific NLRP3 inflammasome activator (Figure 1A) [18]. These results indicate that *P. brasiliensis* can induce the priming but not activation of the inflammasome in BMDMs. As a well-characterized control [32], the cells that were primed with LPS and treated with ATP exhibited high levels of IL-1β production, whereas either LPS or ATP alone did not induce IL-1β secretion (Figure 1A). Conversely, in BMDCs that were infected with *P. brasiliensis* alone, as indicated by the substantial amounts of mature IL-1β, the inflammasome machinery was activated (Figure 2A). Notably, no increments in the IL-1β levels were detected when ATP was added to the infected culture, and LPS alone elicited IL-1β production in BMDCs (Figure 2A). These results suggest that BMDCs differ from BMDMs according to inflammasome activation requirements, which have been recently revealed [33]. As expected, due to significant IL-1β production, high levels of pro-IL-1β were detected in the cell lysate of the BMDCs that were infected with *P. brasiliensis* (Figure 2B). In fact, BMDCs produced nearly two-fold higher pro-IL-1β levels compared with BMDMs (compare Figure 2B with 1B). Additionally, the production of the proinflammatory inflammasome-independent cytokines TNF-α and IL-6 was analyzed. Exposure to the fungus and/or LPS, regardless of the presence of ATP, induced the secretion of these cytokines both in BMDMs and BMDCs (Figure 1C, 1D and 2C, 2D).

### Differential requirement for Myd88- and Syk-dependent signaling in inflammasome priming in BMDMs and BMDCs infected with *P. brasiliensis*

**Figure 3:**
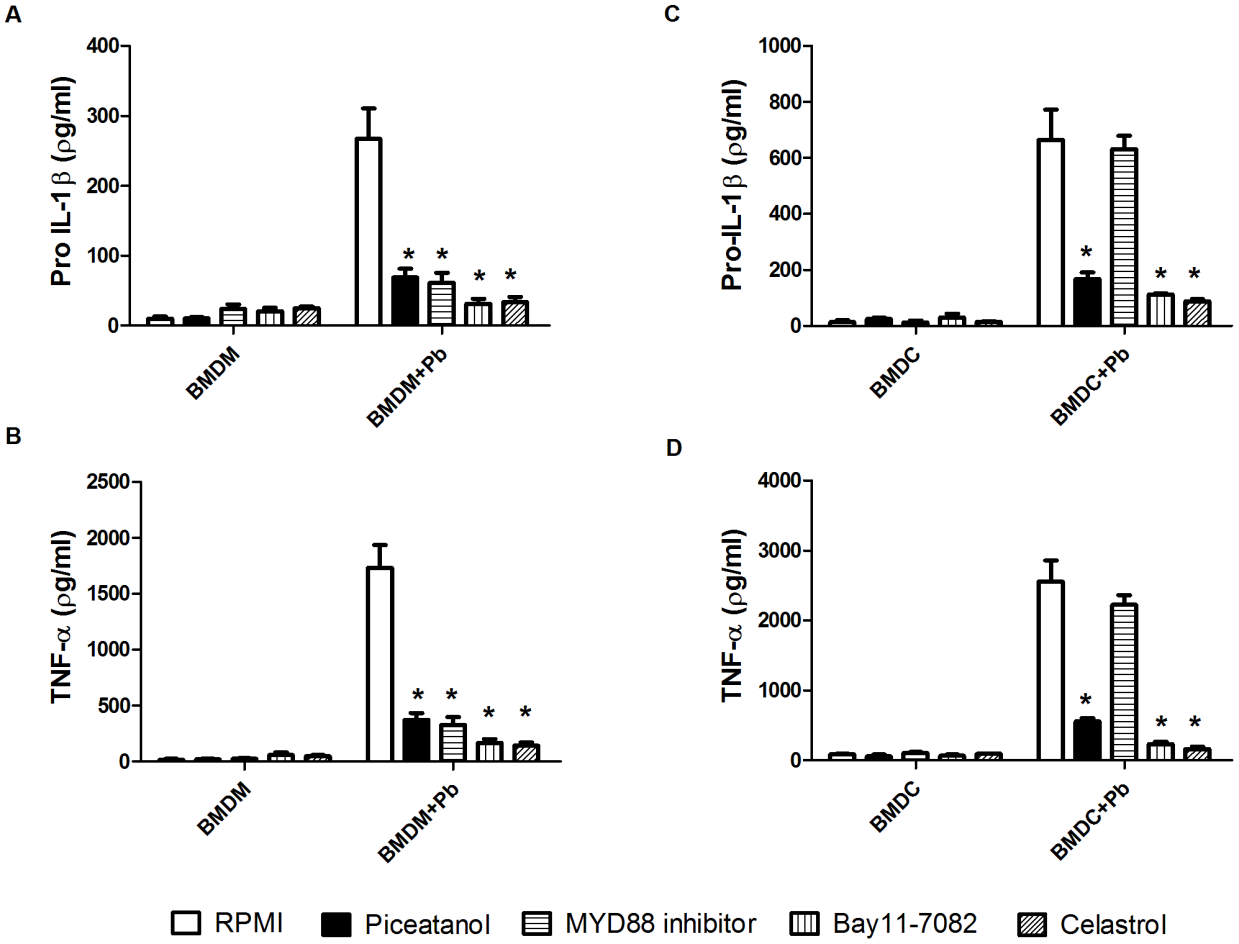
Differential Myd88 and Syk signaling is induced for inflammasome priming in BMDMs and BMDCs infected with *P. brasiliensis*. (A, B) BMDMs and (C, D) BMDCs were pretreated for 1 h with the indicated concentrations (Methods section) of the Syk inhibitor piceatannol, Myd88 inhibitor, and the NFkB inhibitors Bay11-7082 and celastrol. Then, the cells were infected with *P. brasiliensis* (Pb) at an MOI of 1 for 24 h and supernatants and cell lysates were harvested and assayed for TNF-α (supernatants) or pro–IL-1β (lysates) using ELISA. The data are expressed as the mean ± the SD of two to three independent experiments conducted in triplicates. *denotes *p*≤0.05 compared with untreated infected cells.

To clarify the nature of the PRRs that are used by BMDMs and BMDCs to recognize *P. brasiliensis* PAMPs that can induce inflammasome priming (pro-IL-1β production), we pretreated the murine cells with the spleen tyrosine kinase (Syk) inhibitor piceatannol and Pepinh-MYD, a Myd88 signaling inhibitor peptide. After 1 h, the cell cultures were infected with *P. brasiliensis* for an additional 24 h, and pro-IL-1β and TNF-α quantification in the cell lysate and supernatant, respectively, was performed using ELISA (Figure 3). Syk and Myd88 are protein adaptors that are critically important to CLR and TLR signaling pathways, which lead to NF-κB activation and proinflammatory cytokine expression, including pro-IL-1β and TNF-α [8]. Treatment with piceatannol and the Myd88 inhibitor peptide significantly reduced the production of pro-IL-1β and TNF-α in BMDMs. These results indicate the collaboration of these PRRs in the generation of the first signal for inflammasome activation in BMDMs (Figure 3A and 3B). In contrast, only Syk-dependent CLR signaling was required for the production of pro-IL-1β and TNF-α in BMDCs (Figure 3C and 3D). Moreover, we demonstrated that the expression of pro-IL-1β and TNF-α in both cell types depended on NF-κB activity because significantly diminished levels were detected when the cells were pretreated with Bay11-7082, an irreversible inhibitor of IkB-α phosphorylation, which resulted in the inactivation of NF-κB and the NF-κB inhibitor celastrol (Figures 3A, 3B, 3C and 3D). Our results suggest that BMDCs and BMDMs differ according to PRR usage for the production of the proinflammatory cytokines pro-IL-1β and TNF-α and that this process is dependent on NF-κB activation in both cell types as expected.

### NLRP3 inflammasome-dependent activation of caspase-1 is indispensable for IL-1β production by BMDCs infected with *P. brasiliensis*

**Figure 4:**
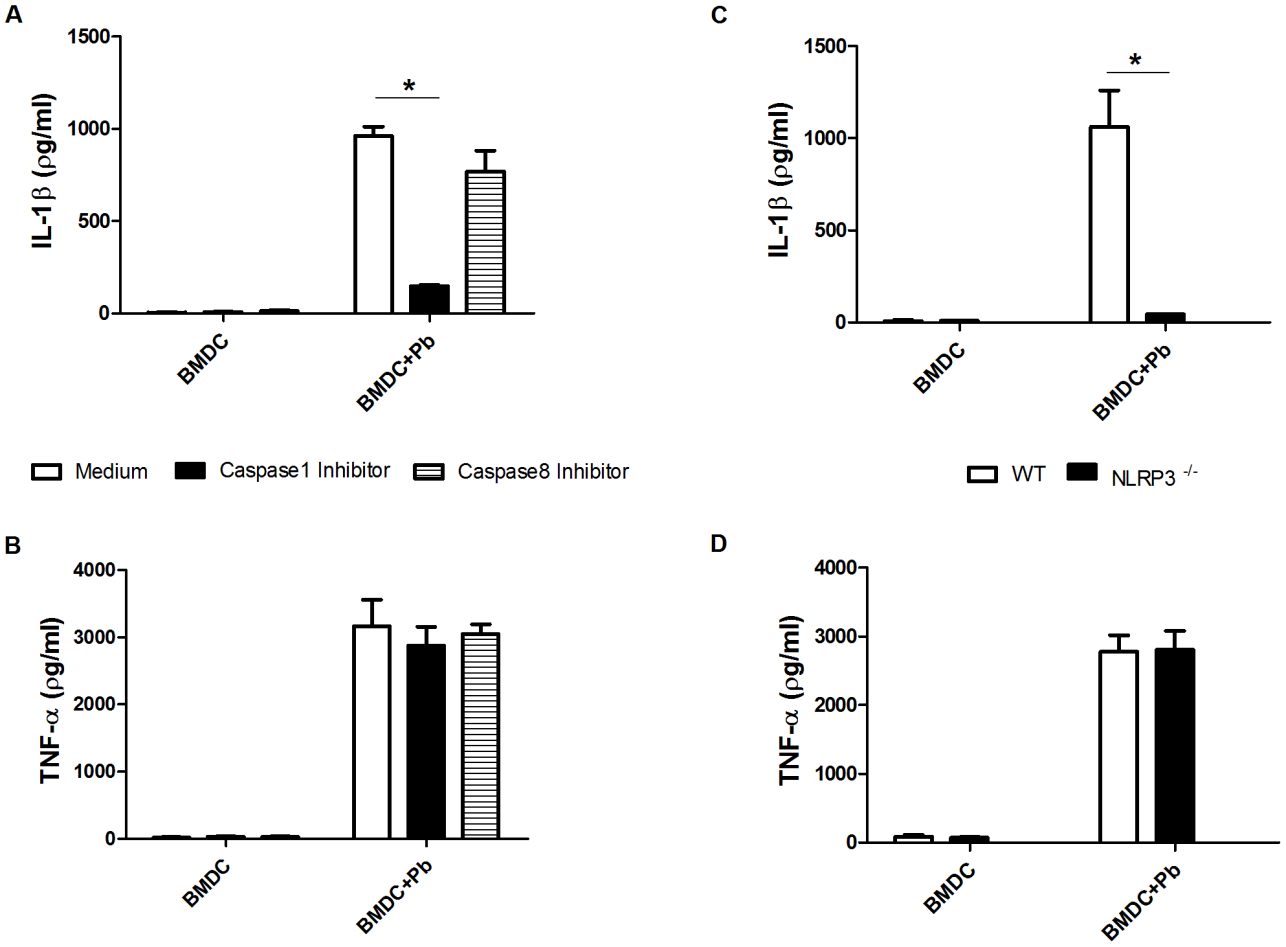
inflammasome activation by *P. brasiliensis* is caspase-1- and NLRP3-dependent. (A, B) BMDCs were pretreated for 1 h with the indicated concentrations (Methods section) of caspase-1 (AC-Y-VAD-CHO) and caspase-8 (Z-IETD-FMK) inhibitors and infected with *P. brasiliensis* at an MOI of 1 for 24 h. (C, D) BMDCs from WT and NLRP3-deficient mice were infected with *P. brasiliensis* (Pb) as above. The supernatants from the cultures were harvested for IL-1β and TNF-α assays using ELISA. The data are expressed as the mean ± the SD of two to three independent experiments conducted in triplicates.*p≤0.05.

Because pro-IL-1β cleavage into mature IL-1β requires active cysteine-aspartic proteases, mainly caspase-1, we selectively inhibited this protease using the aldehyde derivative AC-Y-VAD-CHO in BMDCs that were infected with yeast cells. In addition, the caspase-8 inhibitor Z-IETD-FMK was also used because caspase 8 has recently been associated with the non-canonical processing of IL-1β in DCs infected with *C. albicans* and *A. fumigatus* [34]. IL-1β secretion was abrogated by the caspase-1 inhibitor without affecting inflammasome-independent TNF-α production, whereas caspase-8 activity was not necessary for IL-1β processing in this study (Figure 4A and 4B). IL-1β production in response to diverse fungal pathogens is dependent on the NLRP3 inflammasome; therefore, we evaluated whether *P. brasiliensis*-dependent IL-1β secretion in BMDCs occurred via NLPR3 activation using mice deficient in this protein. As shown in Figure 4C, IL-1β production was almost completely abolished in BMDCs from NLPR3 knockout mice. Thus, IL-1β production by BMDCs infected with *P. brasiliensis* requires NLRP3 inflammasome-dependent activation of caspase-1. As a control, TNF-α production was not affected in mice lacking NLRP3 (Figure 4D).

### K^+^ efflux, ROS production, lysosomal acidification and cathepsin B release are implicated in *P. brasiliensis* NLRP3 inflammasome activation in BMDCs

**Figure 5:**
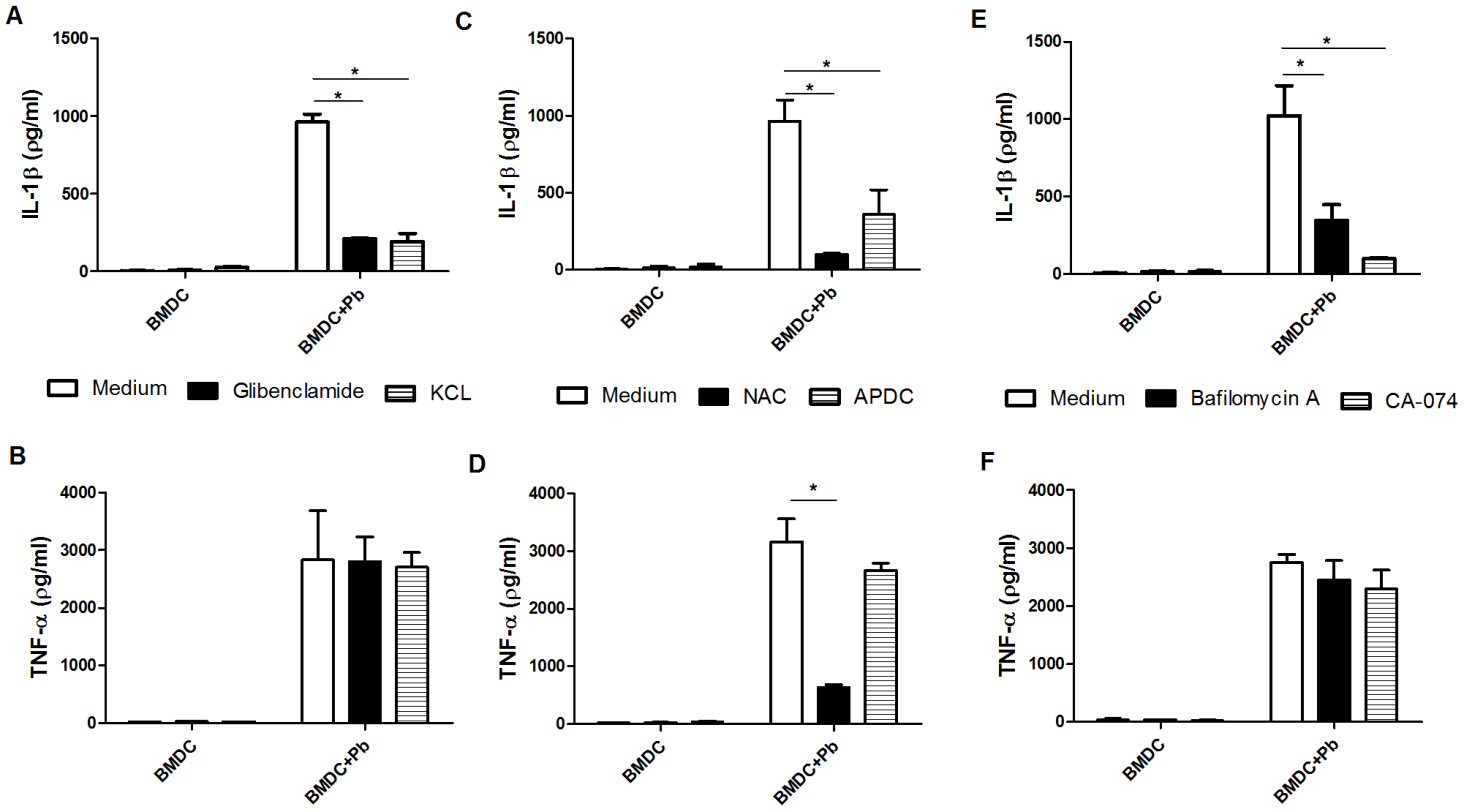
NLRP3 inflammasome activation by *P. brasiliensis* requires K^+^ efflux, ROS production, lysosomal acidification and cathepsin B release. BMDCs were pretreated for 1(Methods section) of (A, B) glibenclamide and KCL, (C, D) NAC and APDC, or (E, F) bafilomycin and CA-074. Then, the cells were infected with *P. brasiliensis* (Pb) at an MOI of 1 for 24 h. The supernatants from the cultures were harvested for IL-1β and TNF-α assays using ELISA. The data are expressed as the mean ± the SD of two to three independent experiments conducted in triplicates. *p≤0.05.

Diverse processes that are associated with intracellular perturbations have been implicated in NLRP3 inflammasome activation, including K^+^ cation efflux, ROS generation, lysosomal acidification and the release of cathepsin B into the cytosol [19]. To study the role of these processes in *P. brasiliensis*-induced inflammasome activation in BMDCs, we initially pretreated the cells with glibenclamide, an ATP-sensitive K^+^ channel inhibitor that precludes the maturation of caspase-1 and IL-1β by inhibiting K^+^ efflux [35]. As shown in Figure 5A, IL-1β processing was significantly reduced by glibenclamide. As an alternative method of blocking K^+^ efflux, we increased the extracellular K^+^ concentration in the culture supernatant, and the secretion of IL-1β was again diminished in BMDCs that were infected with *P. brasiliensis* (Figure 5A). In contrast, the TNF-α secretion levels were unaffected (Figure 5B), which suggests that IL-1β inhibition was not due to any toxic effect on the cells. In addition to K^+^ efflux, the production of ROS, lysosomal acidification and lysosomal damage with the release and activation of cathepsin B into the cytosol have been associated with NLRP3 activation. Therefore, we inhibited ROS using NAC (N-acetyl-L-cysteine) or APDC aminopyrrolidine-2,4-dicarboxylic acid) and also inhibited lysosomal acidification and cathepsin B activity using bafilomycin and CA-074Me, respectively. All the treatments resulted in a significant reduction in IL-1β release from infected BMDCs (Figure 5C and 5E). In contrast, TNF-α secretion was not affected, with the exception of the NAC treatment (Figure 5D and 5F). Indeed, unlike APDC, NAC precludes NF-κB activation [21], [36]. The results indicate that NLRP3 activation in murine DCs that were infected with *P. brasiliensis* requires the efflux of K^+^ coupled with the generation of ROS and lysosomal acidification and damage.

### IL-1β-mediated signaling is required to control the intracellular growth of *P. brasiliensis* in BMDMs

**Figure 6:**
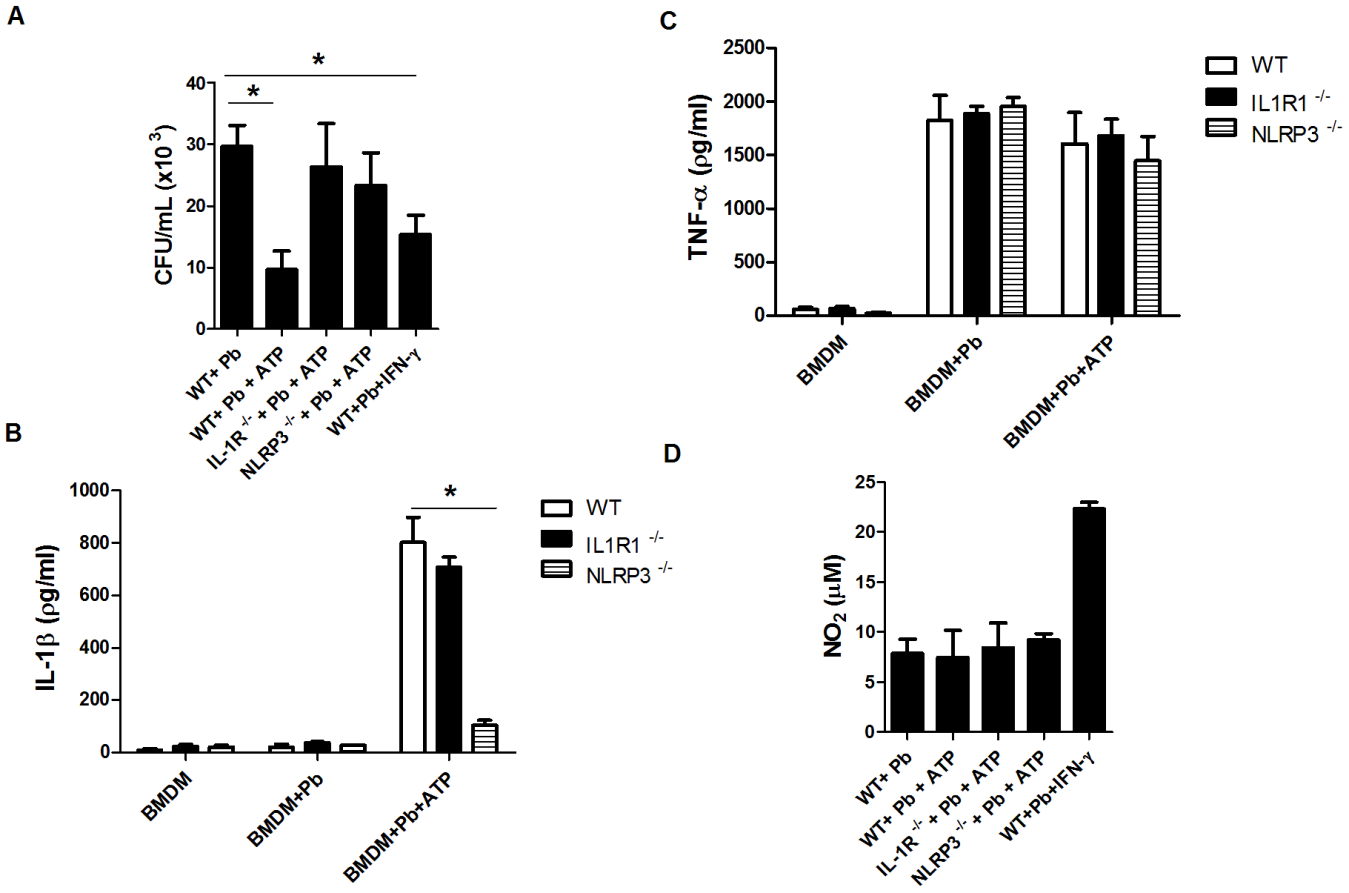
IL-1R1 signaling is required for the anti-fungicidal activity of BMDMs against *P. brasiliensis*. (A) BMDMs were obtained from WT, IL-1R1^−/−^ or NLRP3^−/−^ C57BL/6 mice and were infected with *P. brasiliensis* (Pb) for 24 h at an MOI of 1. As indicated, 1 h before the completion of incubation, 5 mM ATP was added to the culture. After the supernatants were removed, the macrophages were lysed and assayed for the presence of viable yeast cells by CFU assay. (B) IL-1β and (C) TNF-α were detected in the supernatant of infected BMDMs using ELISA. (D) NO_2_ was measured in the supernatant of cell cultures as an indicative of NO production using the Griess assay. The data are expressed as the mean ± the SD of one experiment that was representative of two experiments, which were performed in triplicates. *p≤0.05.

Activated macrophages play a critical role in the restriction of *P. brasiliensis* proliferation *in vitro* and *in vivo*, and IL-1β production has been associated with fungal resistance [7], [21]–[23]. Therefore, we evaluated whether IL-1β-induced signaling is required for the fungicidal mechanism in BMDMs from WT or IL-1R1-deficient mice when infected with *P. brasiliensis* for 24 h. Infected cells were left untreated or were treated with ATP for the induction of IL-1β production via NLRP3 inflammasome activation, as shown in Figure 1. Infected WT BMDMs treated with ATP clearly demonstrated more efficient fungicidal activity, as indicated by the significant reduction in the fungal burden compared with untreated WT macrophages (Figure 6A). As expected, the fungal infection alone did not induce significant IL-1β production, unless ATP treatment was performed (Figure 6B). BMDMs derived from mice lacking IL-1R1 were also unable to restrain fungal growth, despite the significant production of IL-1β induced by ATP treatment (Figure 6A and 6B). Further, NLRP3-deficient BMDMs treated with ATP did not release IL-1β, as expected, and failed to kill internalized fungi (Figure 6A and 6B). Importantly, no different phagocytic ability (data not shown) or TNF-α secretion levels (Figure 6C) was observed in macrophages derived from the different experimental groups. Altogether, these results suggest that IL-1R1 signaling is required for the anti-fungicidal activity of BMDMs against *P. brasiliensis*. To understand the mechanism that was involved in the fungal killing, we measured nitrite as an indicator of NO production in the supernatants of cultures using the Griess reagent. Despite being one of the major products of activated macrophages that are used to kill ingested *P. brasiliensis* [37]–[39], nitrite levels were similar among experimental groups (Figure 6D). Control BMDMs infected with *P. brasiliensis* plus IFN-γ treatment produced significant nitrite levels and fungicidal activity, as previously reported [37], [38] Therefore, other mechanisms must have been activated for the fungicidal activity that was observed.

## Discussion

*P. brasiliensis* induces the transcription and production of pro-inflammatory cytokines via the stimulation of several PRRs. In contrast, inflammasome activation during *P. brasiliensis* infection has never been studied; however, previous works have demonstrated that human monocytes, murine dendritic cells and mice that are infected with this fungus secrete IL-1β, which strongly suggests that an inflammasome must be triggered [27]–[30]. In this study, we found that the NLRP3 inflammasome and caspase-1 are required for the processing and secretion of IL-1β in *P. brasiliensis*-infected BMDCs. Mature IL-1β release depends on the priming and activation of an inflammasome. Using BMDMs and BMDCs, our data suggest that cell-type specificity is involved in the activation of the NLRP3 inflammasome upon fungal exposure. For instance, *P. brasiliensis* can trigger pro-IL-1β production in both bone marrow-derived cell types, but infection resulted in IL-1β maturation and release only in BMDCs. Our results indicate a poor IL-1β response to *P. brasiliensis* in BMDMs, which is in agreement with previous studies that were performed with *C. albicans*. Hise et al. [22] and Joly et al. [23] demonstrated that BMDM IL-1β release required an independent priming step before infection with *C. albicans* to achieve NLRP3 inflammasome activation and a robust IL-1β release. In contrast, BMDCs that were infected with *C. albicans*, *C. neoformans* and *T. schoenleinii* alone release mature IL-1β [21], [24], [25]. This differential regulation of the NLRP3 inflammasome between BMDMs and BMDCs has been attributed to increased NLRP3 protein expression under steady-state conditions in BMDCs [33], [40]. Therefore, after stimulation, the elevated levels of the NLRP3 protein in BMDCs would be sufficient to reach an activation threshold for the NLRP3 inflammasome. *In vivo*, splenic DCs have higher NLRP3 promoter activity when compared with macrophages [41]. In addition, after priming with PRR ligands, NLRP3 and pro-IL-1β protein expression is increased in BMDCs [33]. Similarly, we found that pro-IL-1β production was significantly more evident in BMDCs that were infected with *P. brasiliensis* when compared with BMDMs in this study. The differential regulation of the NLRP3 inflammasome between divergent cell types has been demonstrated in human monocytes and macrophages and in M1- and M2-polarized murine macrophages [42], [43]. Specifically, human monocytes have constitutively active caspase-1, which leads to the release of mature IL-1β after exposure to a single stimuli (e.g., LPS), whereas macrophages utilize the classical two-signal model.

As previously mentioned, several PRRs play a role in the recognition of *P. brasiliensis*, including TLRs (TLR2, TLR4 and TLR9) and the CLR (dectin-1) [10]–[15], which can potentially activate the NF-κB pathway, thereby leading to the production of pro-IL-1β, TNF-α and other pro-inflammatory cytokines. To identify the type of PRR that is associated with this process, before infection with *P. brasiliensis*, we treated BMDMs and BMDCs with inhibitors of the key protein adaptors Myd88 and Syk, which are essential for the majority of TLR and CLR signaling, respectively. BMDMs required both Myd88- and Syk-dependent signaling for the induction of pro-IL-lβ and TNF-α, whereas only the Syk-dependent pathway was necessary in BMDCs. Moreover, regardless of the type of PRR usage, both BMDMs and BMDCs required NF-κB activity, as indicated by the inhibitor assays, to produce IL-1β and TNF-α. These results are consistent with recent findings that TLR ligands and the dectin-1 ligand β-glucan participate in the activation of the NLRP3 inflammasome through a priming effect that is mediated via NF-κB activation [44], [45]. The discrepancy between macrophages and dendritic cells regarding PRR usage is consistent with studies that demonstrated dectin-1 Syk-dependent signaling alone is not sufficient to induce TNF-α production in BMDMs in contrast to BMDCs because synergizing with the Myd88 pathway is required for the production of this cytokine [46]. This unresponsiveness in macrophages, when compared with dendritic cells, is associated with the differential use of the caspase recruitment domain (CARD)-containing adaptor protein CARD9 by dectin-1 receptor [47]. Similar to the results in the present study, pro-IL-1β and TNF-α production that was induced by *C. albicans* in BMDCs [21] was Syk-dependent and Myd88-independent in contrast to macrophages [22]. In addition, the human acute monocytic leukemia cell line (THP-1) required signaling through Syk and Myd88 for inflammasome priming when infected with *A. fumigatus* [26]. In this context, we recently found that the transcript levels of genes that encode TLRs and adaptor proteins (e.g., Myd88) were not increased in *P. brasiliensis*-infected BMDCs, suggesting a minor role for the TLR pathway in the induction of cytokines, at least in dendritic cells [14]. In contrast, dectin-1 transcripts were highly abundant, which suggests that this receptor plays an important role in the recognition of *P. brasiliensis* PAMPs (i.e., β-glucan) by dendritic cells. It remains to be investigated whether a deficiency in specific TLRs (e.g., TLR2/4/9) or CLRs (e.g., dectin-1) prevents inflammasome activation by *P. brasiliensis*.

The maturation of IL-1β requires proteolytic activity of inflammatory caspases. In addition to the canonical caspase-1-dependent inflammasome, caspase-8 has been recently associated with the non-canonical processing of IL-1β in BMDCs that were infected with *C. albicans* and *A. fumigatus* [34]. Therefore, we performed inhibitor assays using either AC-Y-VAD or Z-IETD-FMK, which are specific inhibitors of caspase-1 and caspase-8, respectively. Caspase-1 activity was necessary for *P. brasiliensis*-induced IL-1β release in BMDCs. In contrast, caspase-8 was not required, which was also demonstrated in BMDCs that were infected with *C. neoformans* [25]. This finding indicates that a specific signaling pathway may direct IL-1β induction by different fungi. Notably, caspase-8 has diverse activities that suppress innate immunity, including the antagonistic function of restricting NLRP3 inflammasome activation [48]. Caspase-1 activation is mainly dependent on the assembly of inflammasomes. Regarding fungal infections, the NLRP3 inflammasome is essential for casapase-1 activity and subsequent IL-1β release in murine and human phagocytes that are infected with *C. albicans*, *A. fumigatus*, *T. schoenleinii* and *C. neoformans* [21]–[26]. A similar finding was obtained for *P. brasiliensis*-infected BMDCs because there was an obvious abrogation of IL-1β release from NLRP3 knockout cells, which did not affect TNF-α production. We did not evaluate ASC function in this study; however, our results strongly suggest that caspase-1-dependent NLRP3 inflammasome is essential for the release of IL-1β by BMDCs that are infected with *P. brasiliensis*.

No direct mechanistic studies have been published; however, several intracellular perturbations and danger signals have been demonstrated to lead to the activation of the NLRP3 inflammasome, including K^+^ efflux, ROS generation, lysosomal acidification and cathepsin B release to the cytosol [19]. K^+^ efflux is required for NLRP3 inflammasome activation in BMDCs that are infected with *C. albicans*, *T. schoenleinii* and *C. neoformans* [21], [24], [25]. To test whether K^+^ efflux plays a role in inflammasome activation in BMDCs that are infected with *P. brasiliensis*, the cells were pretreated with glibenclamide, or the extracellular K^+^ concentration in the culture supernatant was increased. Both treatment approaches impaired IL-1β release without interfering with TNF-α secretion, which indicates that reduced IL-1β production was not due to treatment-induced cell death. Comparable results were obtained when we used APDC, an inhibitor of the NADPH oxidase-dependent ROS system, suggesting a role of ROS in *P. brasiliensis*-induced NLRP3 inflammasome activation. In this context, *P. brasiliensis* infection leads to ROS production via NADPH oxidase in phagocytes [49]–[51]. Regarding the antioxidant or free radical scavenger NAC, a concomitant reduction in both IL-1β and TNF-α secretion was observed, which is in line with the finding that NAC, unlike APDC, inhibit NF-κB activation [21], [36]. The prevention of lysosomal acidification acts as a NLRP3 inflammasome inhibitor, which suggests that acid-dependent lysosomal thiol proteases have a role in NLRP3 activation [52], [53]. Therefore, BMDCs were treated before fungal infection with bafilomycin, which inhibits the vacuolar H^+^ ATPase or CA-074, which inhibits cathepsin B activity. Both lysosomal acidification and cathepsin activity play a role in *P. brasiliensis*-induced IL-1β release with no interference in TNF-α production. Similar to the activation of the inflammasome by particulate matters, bacteria and fungi [54]–[56], [23]–[25], this result suggests that *P. brasiliensis*-induced NLRP3 inflammasome assembly may be linked to lysosomal damage. Altogether, we demonstrate that the NLRP3 inflammasome activation induced by *P. brasiliensis* infection required common intracellular perturbations, which are triggered by numerous stimuli, including fungi.

*P. brasiliensis* acts as a facultative intracellular pathogen in non-activated human and murine macrophages and can survive and replicate within these cells [7]. In this context, for PCM and other systemic mycoses, such as cryptococcosis and histoplasmosis, fungal intracellular parasitism has been proposed to be a major event for disease establishment and progression in susceptible hosts, which enables fungal latency and dissemination from the primary infected organ to other organs [9], [57]. To assess whether IL-1β-mediated signaling plays a part in the activation of macrophage fungicidal activity, BMDM cultures from WT or IL-1R1^−/−^ mice were infected with *P. brasiliensis*, and a CFU analysis was performed. We demonstrated that BMDMs do not produce IL-1β in response to *P. brasiliensis* infection; therefore, we treated the infected cells with ATP for the robust release of this cytokine via NLRP3 inflammasome activation. Interestingly, WT BMDMs that were treated with ATP, which produce and respond to IL-1β, had a significant reduction in the fungal burden compared with WT BMDMs that were not treated with ATP. In addition, despite IL-1β production in IL-1R1^−/−^ BMDMs that were treated with ATP, fungal proliferation was not constrained in these cells. These results suggest that IL-1β signaling is an important activator of the microbicidal activity of BMDMs against *P. brasiliensis*. This mechanism is not associated with the induction of nitric oxide production, a potent *P. brasiliensis* fungicidal molecule [37]–[39] because no differences in the NO_2_ supernatant levels were observed. Therefore, other microbicidal mechanisms may be activated. In fact, IL-1R1 signaling is required for the enhancement of phagolysosomal maturation (i.e., phagosome-lysosome fusion and acidification) and microbicidal activity of macrophages infected with *Mycobacterium tuberculosis* [58]–[60]. In fungi, efficient killing of *C. albicans* and *Histoplasma capsulatum* requires phagolysosomal maturation, independent of nitric oxide function [61]–[63].

IL-1β-mediated signaling has been implicated in controlling several intracellular bacteria [60], [64], [65]. Regarding fungi, IL-1R1-deficient mice were severely impaired in both innate and adaptative responses in a disseminated model of candidal infection, including a lack of antifungal killing activity in neutrophils against fungal yeast [66]. Consistent with these results, mice deficient in NLRP3 that were intravenously infected with *C. albicans* had reduced serum IL-1β levels, decreased survival and a higher fungal burden in several organs [21]. In addition, IL-1R1, NLRP3 and the inflammasome components ASC and caspase-1 were necessary to block local mucosal colonization and the systemic dissemination of *C. albicans* in a murine oral model [22]. Regarding *C. neoformans*, mice that lacked NLRP3 or ASC and were infected through intraperitoneal or intranasal routes had significantly reduced survival [25]. Therefore, future studies must be conducted to address whether IL-1R1 and NLRP3 inflammasome components play a protective role against *P. brasiliensis* infection *in vivo* and to determine how IL-1β activates macrophages for enhanced killing activity *in vitro*.

In summary, the infection of murine dendritic cells with the primary fungal pathogen *P. brasiliensis* primes, via Syk signaling, and activates the caspase-1-dependent NLRP3 inflammasome. The activation process involves K^+^ cation efflux, ROS production, lysosomal acidification and active cathepsin B for IL-1β processing and release. Moreover, macrophages require IL-1β for antifungal killing activity. These results suggest that manipulating NLRP3 inflammasome activation may provide a new approach for the control of PCM.
